# Glycothymoline as an Oronasal and a General Antiseptic

**Published:** 1905-05

**Authors:** 


### ABSTRACTS.

---

### Glycothymoline as an Oronasal and a General Antiseptic.

DAVID WALSH, Senior Physician to Western Skin Hospital, London (*Medical Press and Circular*), remarks that it is only by slow degrees that medical men as a profession are learning to realise the important part played by bacteria in the cavities of the nose and mouth. One sign of this appreciation may be found in the fact that washes and gargles for the mouth and throat are being more and more adopted in everyday practice. The systematic use of such applications, however, so far as the nostrils are concerned, is for the most part still confined to specialists. One reason for this comparative neglect of a simple method of treatment by general practitioners has, no doubt, hitherto lain in the difficulty of obtaining a safe and at the same time, an efficient antiseptic and cleansing fluid for the mucous membranes in question.

Glycothymoline was brought to my notice lately as an excellent lotion for nasal and oral sprays and washes. On due inquiry it was found to fulfill the conditions usually recognised by medical men in the United Kingdom as vouching for the character, so to speak, of such a preparation; its composition is not a secret, its formula being freely published. Under these circumstances, I determined to try the effect of this preparation in a few suitable cases. As a general antiseptic fluid that does not coagulate albumin and is nonirritant, deodorant and practically nonpoisonous, glycothymoline has clearly a wide range of usefulness. My own observations, however, have been practically limited to its use in the nose and mouth, with results that have proved satisfactory in every instance, especially in acute coryza, pharyngitis, influenza and septic conditions of the mouth.

In glycothymoline we have a good and safe application in all septic conditions of the mouth, throat and nose. It seems not improbable that in the near future medical men will attend more than they have done hitherto to the mucous membranes of the upper respiratory tract in influenza, measles, scarlatina, chronic and acute coryza, whooping-cough and other infectious ailments. Postnasal catarrh—that plague of modern civilisation—has never been adequately attacked by the general practitioner. Carious teeth, again, another defect of civilisation, are apt to damage the general health considerably. In both these conditions glycothymoline will be found a safe and effective remedy well worth a careful trial in practice.

